# Intraocular Metastasis: Differential Diagnosis and Management

**DOI:** 10.1111/ceo.70100

**Published:** 2026-03-07

**Authors:** Genovefa Μachairoudia, Dimitrios Kazantzis, Guy S. Negretti, Mandeep S. Sagoo

**Affiliations:** ^1^ Ocular Oncology Service Moorfields Eye Hospital London UK; ^2^ Retinoblastoma Service Royal London Hospital London UK; ^3^ Medical Retina Service Moorfields Eye Hospital London UK; ^4^ NIHR Biomedical Research Centre for Ophthalmology at Moorfields Eye Hospital and UCL Institute of Ophthalmology London UK

**Keywords:** choroidal metastasis, intraocular metastasis, radiotherapy, uveal metastasis

## Abstract

Intraocular metastases represent the most common type of malignant intraocular tumour in adults. These commonly affect the choroid but can also involve the iris, ciliary body, retina, vitreous, optic disc or lens. Breast and lung cancer are the most common origins of intraocular metastases. The diagnosis of intraocular metastases can be particularly challenging in patients without a prior history of cancer. Furthermore, treatment decisions may be complex, especially in individuals already receiving systemic therapy or in those with previous ocular treatment history. Herein, we present a review of the current knowledge regarding the epidemiology, origins, clinical and imaging characteristics, differential diagnosis and treatment of intraocular metastases.

## 1 Introduction

Intraocular metastases represent the most common type of malignant intraocular tumour in adults, often posing significant diagnostic and therapeutic challenges for both ophthalmologists and oncologists [1]. The eye, with its rich vascular supply, especially within the choroid, is a frequent site of metastatic cancer deposits, most commonly originating from primary malignancies in the breast and lung [1]. The clinical presentation is often subtle, mimicking a variety of benign and malignant ocular conditions, which complicates timely diagnosis and management. With improvements in cancer therapies leading to longer patient survival, recognition and management of intraocular metastasis have gained increasing clinical importance.

The aim of this review is to present a comprehensive and detailed overview of intraocular metastasis with a focus on the differential diagnosis and management. By synthesising current evidence and clinical practice, the review seeks to enhance understanding of the epidemiology, clinical presentation, diagnostic challenges, and therapeutic options for intraocular metastatic disease. Ultimately, it aims to support clinicians in timely identification and effective multidisciplinary management of patients, thereby improving visual outcomes and quality of life for those affected by this significant complication of systemic cancer.

## 2 Epidemiology and Origin

The reported incidence of intraocular metastases varies considerably across studies, largely reflecting differences in methodologies and patient populations. Early histopathological analysis by Albert et al. (1967) identified choroidal metastases in 5 out of 213 patients with confirmed metastatic cancer, corresponding to an incidence of approximately 2.3% [2]. Subsequently, Nelson et al. conducted a prospective histopathological study involving 716 patients with malignant neoplasms and found intraocular metastases in 52 cases, yielding a higher incidence of 7.3% [3]. Similarly, Eliasi‐Rad et al. examined 741 eyes from the Wisconsin Eye Bank and the ocular pathology records at the Wilmer Eye Hospital and reported that while only 1%–4.7% of eyes exhibited grossly visible intraocular metastases, histopathological evaluation detected metastases in as many as 12.6% of eyes [4]. These findings demonstrate that clinical detection rates tend to underestimate the true prevalence of intraocular metastases. This discrepancy is primarily due to the frequent asymptomatic nature of ocular metastases and the lack of systematic ophthalmic screening in cancer patients. Autopsy and histopathology‐based studies reveal a substantial proportion of subclinical lesions.

Due to the rich vascular supply, which facilitates the hematogenous dissemination of tumour cells, the choroid is the most common anatomical site for intraocular metastasis, accounting for approximately 88%–90% of all cases identified [1]. Shah et al. found that the choroid was involved in 88% of 374 uveal metastatic tumours originating from primary lung cancer, and Demirci et al. reported choroidal metastases in 85% of 264 consecutive patients with uveal metastases from breast cancer [5, 6]. The choroid's extensive network of capillaries provides a favourable microenvironment for metastatic tumour emboli to be deposited and proliferate [7]. The iris and ciliary body represent a smaller proportion of intraocular metastases, comprising approximately 8% and 2% of intraocular metastases [1, 8]. Metastatic involvement of other intraocular sites includes the retina, optic disc, vitreous and lens capsule, and is exceedingly rare [8]. Due to their relatively limited vascularisation compared to the choroid, these areas are less prone to hematogenous dissemination of tumour cells.

The epidemiological and clinical features of intraocular metastases, as reported in major studies, are presented in Table 1. A retrospective review of 2214 uveal metastases of 1111 patients in Wills Eye Hospital during a 44‐year period showed a mean age of 60 years and a female predominance [9]. This can be attributed to the more frequent occurrence of metastatic breast cancer to the uvea in female patients. The most common primary site was breast cancer (37%) followed by lung cancer (27%) and kidney cancer (4%). The presentation was unilateral in 82% of uveal metastases. A Chinese cohort reported a younger mean age (54 years), no gender predominance, and lung cancer as the most common primary (49%), with unilateral disease in 86% [10]. However, in both studies, patients with breast cancer as primary malignancy were diagnosed at a younger age compared to patients with uveal metastases secondary to lung cancer [9, 10]. An analysis of iris metastases by Liu et al. showed a mean age of 56 years, male predominance, and lung and breast cancer as the most frequent primaries [11]. The differences between the above studies may partly be explained by differences in smoking rates worldwide.

**TABLE 1:** Summary of epidemiological and clinical features of intraocular metastases reported in major studies.

Study	Region	Patients/Eyes	Mean age (years)	Gender predominance	Most common primary cancers (%)	Predominant ocular site	Unilateral (%)	Primary diagnosed after ocular Dx (%)	Primary never identified (%)	Survival findings
Shields et al. (Primary tumour origin‐based analysis) [9]	USA	1111 pts./1310 eyes/2214 lesions	60	Female	Breast (37%), lung (27%), kidney (4%)	Uvea (predominantly choroid)	82%	17% (lung carcinoma, lung carcinoid, pancreatic cancer)	16%	Median OS: 17.2 months; favourable 5‐year OS in lung carcinoid (92%)
Li et al. [10]	China	161 pts./185 lesions	54	None	Lung (49%), breast (22%), kidney (4%)	Uvea (predominantly choroid)	86.30%	24.20%	NA	Median OS: 18.84 months
Liu et al. [11]	Multicentre	125 pts./133 eyes	56	Male	Lung (42%), breast (15%)	Iris only	88%	NA	5%	Average survival time: 7 ± 9 months
Welch et al. (sex‐based analysis) [12]	USA	1111 pts./1310 eyes/2214 lesions	63 (M)/58 (F)	Female	F: Breast (58%), M: lung (40%)	Uvea (predominantly choroid)	89% (M)/79% (F)	NA	F: 11%, M: 21%	Median OS: 12.6 months (M) vs. 19.8 months (F); 5‐year OS: 21% (M) vs. 31% (F)
Welch et al. (timing of primary cancer diagnosis—based analysis) [13]	USA	1111 pts./1310 eyes/2214 lesions	59 (primary known)/61 (primary diagnosed later)	Female (primary known)/none (primary diagnosed later)	Primary known: breast (35%), lung (14%), others (18%)	Uvea (predominantly choroid)	79% (primary known)/89% (primary diagnosed later)	17% (lung carcinoma, lung carcinoid, GI tract, pancreatic cancer)	16% of all cases—48% of unknown‐primary cases	No difference in 5‐year OS by primary status

Most patients with intraocular metastases have a known primary malignancy at diagnosis (67%–76%) [9, 10]. Nevertheless, it is significant that in approximately one‐quarter to one‐third of cases, the discovery of intraocular metastatic lesions leads to the identification of a previously undetected primary tumour. Among patients with unknown primary tumours, nearly half never have a primary cancer identified [13]. Importantly, 5‐year overall survival does not differ between patients with known primary tumours, those diagnosed after ocular metastasis, or those in whom the primary malignancy remains unidentified [13].

## 3 Clinical Presentation and Differential Diagnosis

### 3.1 Choroidal Metastasis

The clinical presentations of choroidal metastases are related to their impact on vision and retinal anatomy. Most patients present with blurred vision, but symptoms can also include photopsiae, floaters, visual field defects, and sometimes ocular pain [14, 15, 16]. A significant minority of patients with choroidal metastases may be asymptomatic, with an incidental finding during routine examination [14]. A report from the Liverpool Ocular Oncology service reported that blurred vision is the most common symptom in 55.2% of patients and that 13.5% of patients with choroidal metastases were asymptomatic [14].

Choroidal metastases typically appear on clinical examination as yellowish plateau‐shaped subretinal masses with indistinct borders [17]. The colour of a choroidal metastasis may help distinguish the origin of the primary cancer. For example, Shields et al. (2018) reported that 63% of metastases arising from cutaneous melanoma were brown while metastases arising from other cancers were brown in only 0%–3% [9]. Red‐orange colour might also be a feature suggestive of cancers arising from kidney or thyroid carcinomas. Salcedo‐Villanueva et al. described that in a Mexican cohort of patients with choroidal metastases, all three patients with choroidal metastases from renal cell cancer presented with a red‐orange colour and Haimovici et al. reported that a reddish‐orange appearance of the tumour was present in two out of five patients with renal cell carcinoma [18, 19]. Macular involvement is common and may lead to significant visual loss. Serous retinal detachment is a common feature and can be observed as subretinal fluid adjacent to or overlying the choroidal metastasis [18].

Imaging is fundamental to both the diagnosis and the differentiation of choroidal metastases from other choroidal lesions. Optical coherence tomography (OCT) typically reveals a dome‐shaped or undulating elevation of the choroid, with irregularity or thickening of the overlying retinal pigment epithelium [20]. Enhanced depth imaging (EDI)–OCT can highlight irregularity (lumpy, bumpy appearance) of the anterior surface of the tumour, a pathognomonic feature of choroidal metastases, and compression of underlying choroidal structures [21]. OCT can identify the presence and extent of subretinal fluid [22]. It can also distinguish the presence of lipofuscin and hyperreflective dots (‘speckles’) [23]. The inner retinal layers are usually intact. Studies using OCT‐angiography have demonstrated thinning of the choriocapillaries, a disorganised choroidal vascular plexus and the absence of intratumor vasculature, which may help differentiate metastases from choroidal melanomas, which typically have intratumor vasculature [24, 25].

Fundus autofluorescence (FAF) imaging can show a hypoautofluorescent lesion due to loss of retinal pigment epithelium fluorescence or hyperautofluorescence, which correlates with hyperpigmentation and subretinal fluid or presence of lipofuscin [26, 27].

**FIGURE 1:**
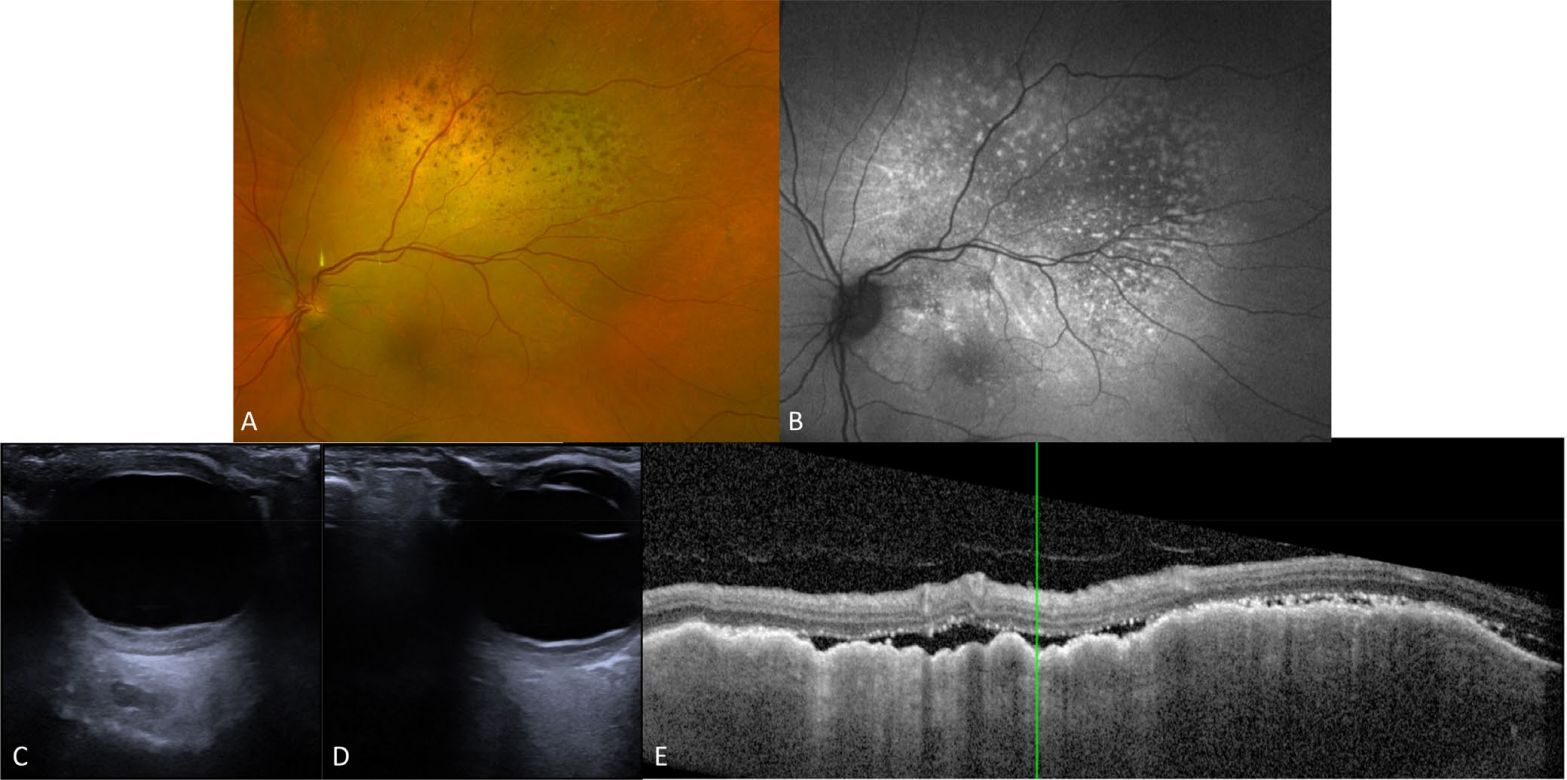
Choroidal metastasis secondary to lung cancer. On pseudocolour (A) and autofluorescence (B) images, there is a yellowish choroidal lesion with overlying lipofuscin. On high resolution ultrasound (C and D), it has medium internal echogenicity, with transverse base 12.8 mm, longitudinal base 14.6 mm and elevation 1.1 mm. The OCT scan (E) shows choroidal thickening with lumpy bumpy appearance and overlying subretinal fluid and trace of lipofuscin. Patient consent obtained for the use of images for publication.

On ultrasonography, choroidal metastases typically demonstrate moderate to high internal reflectivity on A‐scan ultrasonography, a feature that helps distinguish them from choroidal melanomas, which more often exhibit lower internal reflectivity [28]. On B‐scan ultrasound, choroidal metastases appear as echogenic, plateau‐shaped lesions with a broad, shallow configuration and a low height‐to‐base ratio [29]. Subretinal fluid overlying or adjacent to the metastatic lesion may be observed on B‐scan. Tumour thickness measured by ultrasonography may correlate with the primary cancer site, with metastases from cutaneous melanomas typically exhibiting the smallest thickness (< 2 mm) and those from kidney cancer showing greater thickness (approximately 3.8 mm) [9] (Figure 1).

Fluorescein angiography (FA) typically demonstrates early hypofluorescence followed by late, non‐homogeneous hyperfluorescence, whereas indocyanine green angiography reveals hypocyanescence with a subtle, blurred visualisation of the normal choroidal pattern through the tumour [30, 31].

On magnetic resonance imaging (MRI), choroidal metastases typically appear isointense on T1‐weighted images and hypointense on T2‐weighted images [32]. In a recent retrospective study of 42 patients with confirmed choroidal metastases, Yu et al. reported an MRI sensitivity of 50% based on routine clinical interpretation. However, it improved to 76% with increased utilisation of fat‐suppressed fluid‐attenuated inversion recovery (FLAIR) imaging and detailed ocular scrutiny [33].

In the setting of suspected choroidal metastases without an identified primary malignancy, two approaches are possible: systemic imaging under the supervision of a medical oncologist or biopsy of the ocular deposit. The method of tumour biopsy depends on the site of metastasis. For choroidal metastases, two main approaches are used: transscleral and transvitreal. The choice between these depends on the location and size of the tumour, as well as the surgeon's preference and expertise, with the transscleral approach generally reserved for anterior tumours and the transvitreal approach for smaller and more posterior tumours.

#### 3.1.1 Transscleral Biopsy Technique

Initially, following conjunctival peritomy, the tumour is located using transillumination, indirect ophthalmoscopy, or ultrasound. A partial thickness triangular or rectangular scleral flap over the thickest part of the tumour (10 mm × 5 mm) is created. At the base of the flap, a 3 mm full thickness incision is made into the choroid. We use Essen biopsy forceps (Dutch Ophthalmic Research Center, Zuidland, The Netherlands) to take 2–4 samples from the tumour. In our centre, we scrape the cells/tissue sample onto a small piece of cardboard and add the mounted sample to a 10% formalin solution in a sterile container. Any cells that have extruded through the incision are diathermied and then the flap is glued back into place and 10‐0 Nylon sutures placed if required.

#### 3.1.2 Transvitreal Biopsy Technique

Transvitreal biopsy can be performed using either fine‐needle aspiration biopsy (FNAB) or vitreous cutter biopsy, with the choice determined by equipment availability and surgical expertise. FNAB is typically performed with a 25–30‐gauge needle, which has a bevel length of 0.8–1.5 mm and a sharp tip. Vitreous cutters, in contrast, are usually 25‐ or 27‐gauge instruments with a blade‐to‐apex length of 0.23–0.30 mm and a flat base, allowing safe sampling of very small lesions with minimal risk of perforation compared with a sharp needle.

The transvitreal choroidal biopsy is performed using a standard 3‐port vitrectomy setup. A vitrectomy is not required; the cutter is connected to a vacutainer or aspirated manually with a syringe. The cutter is introduced directly into the lesion until contacting the sclera, infusion pressure is raised, and the cutter is rotated in both directions while cutting and aspirating. Intraocular pressure is gradually lowered before withdrawing the cutter to minimise bleeding. The samples, mounted by scraping the tissue onto cardboard, are placed in an equal volume (1:1) of 10% formalin. All ports are sutured, and triple freeze–thaw cryotherapy is applied to the sclerotomies.

The main disadvantage of the transvitreal approach is that specialist cytopathology is not widely available in ophthalmic centres, and limited expertise increases the risk of non‐diagnostic biopsy results. However, in centres with appropriate expertise, this technique offers the advantage of safely biopsying small lesions, including those in the posterior pole or adjacent to the optic nerve, with low complication rates [34, 35].

The differential diagnosis of choroidal metastases is broad and includes a wide range of choroidal tumours (such as melanoma, osteoma, and hemangioma), choroidal infectious diseases (including abscesses and granulomas), subretinal haemorrhage, posterior scleritis, solitary idiopathic choroiditis, and inflammatory conditions such as Vogt–Koyanagi–Harada disease [1, 8]. Table 2 presents the key imaging features that help distinguish these entities [14, 15, 16, 17, 18, 19, 20, 21, 22, 28, 29, 30, 31, 32, 36, 37, 38, 39, 40].

**TABLE 2:** Clinical characteristics and imaging features of choroidal lesions that mimic choroidal metastasis.

Imaging modality	Choroidal metastasis	Choroidal melanoma	Choroidal osteoma	Choroidal hemangioma	Posterior scleritis	Choroidal granuloma
Fundoscopy	Yellowish, plateau‐shaped, ill‐defined borders	Dome‐ or mushroom‐shaped, pigmented or amelanotic, distinct margins	Yellow‐white, well‐demarcated, near disc	Orange‐red, well‐circumscribed, solitary lesion	Choroidal folds, possible subretinal fluid, disc swelling, rarely nodular	Yellowish‐white, elevated, indistinct borders, usually single—can be multiple
OCT	Dome‐shaped or undulating elevation (lumpy‐bumpy), irregular RPE, subretinal fluid	Dome‐shaped elevation, optical shadowing, presence of lipofuscin	Irregular choroidal thickening, lamellar bone structure visible, marked shadowing	Smooth dome‐shaped expansion, choroidal vessels visible, often overlying subretinal fluid	RPE irregularity, subretinal fluid, choroidal thickening	Homogenous, hyporreflective choroidal thickening, dome‐shaped elevation, increased signal transmission, subretinal fluid
Ultrasonography	Moderate‐high reflectivity (A‐scan), shallow, broad‐based mass, low height‐to‐base ratio	Low‐medium reflectivity (A‐scan), mushroom‐shaped, higher height‐to‐base ratio	High reflectivity, dense echogenic lesion, strong shadowing	High internal reflectivity; echogenic dome with low shadow	Scleral thickening, rarely a nodule, ‘T‐sign’ representing fluid in Tenon's space	Hyperechoic choroidal mass, low‐to‐moderate reflectivity
Fluorescein angiography	Early hypofluorescence, late hyperfluorescence with pinpoint leakage	Early hypofluorescence, late staining/leakage, ‘double circulation’ pattern	Early patchy hyperfluorescence, late staining	Early hyperfluorescence, progressive late staining	Variable leakage, disc oedema, and possible other inflammatory signs such as retinal vascular leakage	Early hypofluorescence, late hyperfluorescence, leakage into lesion and around subretinal fluid, other inflammatory signs also possible
ICG Angiography	Hypocyanescence with minimal late staining	Tumour vascularity/double circulation, late hypercyanescence	Blocked or variable, often reduced by calcification	Early, intense hypercyanescence	Usually normal or mild hypercyanescence	Hypocyanescence
MRI	Isointense on T1, hypointense on T2	Hyperintense on T1, hypointense on T2	Hypointense on both T1 and T2 due to calcification	Hyperintense on T1 and T2	Scleral thickening, enhancement, periscleral inflammation	Iso‐ to hypointense on T1, hypointense on T2, may reveal solid or inflammatory mass

### 3.2 Iris Metastasis

An iris metastasis typically presents with symptoms such as blurred vision, ocular pain, photophobia, or redness, often due to mass effect or secondary complications such as elevated intraocular pressure [41]. On clinical examination, these most commonly appear as solitary or multiple yellow‐white, pink, or occasionally pigmented nodules on the iris stroma. These lesions may be associated with neovascularisation, anterior chamber cells or flare, hyphaema, irregular pupil shape, and secondary glaucoma [42] [Correction added on 23 March 2026, after first online publication: The term “hyphaemia” has been corrected to “hyphaema” in Section 3.2 Iris Metastasis and Table 3.]. Anterior segment OCT will show a thickening and an irregular anterior surface and nodules, while ultrasound biomicroscopy (UBM) will reveal homogenous moderate reflectivity with internal hyporeflective spaces [43, 44].

Liu et al. retrospectively analysed 133 metastatic iris tumours and reported that the most common primary tumours were lung cancer (42%) and breast cancer (15%) [11]. In approximately one‐third of the cases, iris metastases were the first clinical manifestation prompting the diagnosis of an underlying malignancy [11].

The differential diagnosis of an iris metastasis is broad and includes primary iris melanoma (especially amelanotic types), benign iris cysts, granulomatous diseases such as sarcoidosis and tuberculosis, iris lymphoma, juvenile xanthogranuloma and inflammatory conditions such as Fuchs' heterochromic iridocyclitis [45]. Clinical context and demographic parameters, careful ophthalmic examination, imaging features, and when necessary cytological or histopathological analysis are helpful for distinguishing metastatic deposits from other iris lesions. Table 3 highlights distinguishing features in clinical presentation and imaging findings differentiating iris metastasis from other iris lesions [11, 41, 42, 43, 44, 45, 46, 47].

**TABLE 3:** Clinical and imaging features differentiating iris metastasis from other iris lesions.

Feature	Iris metastasis	Iris melanoma	Iris cyst	Iris lymphoma
Clinical presentation	Pain, redness, blurred vision, irregular pupil, hyphaema	Often asymptomatic or visible pigmented mass, may cause pupil distortion	Usually asymptomatic, smooth translucent lesion	Painless, diffuse iris thickening, red eye
Colour/appearance	Yellow‐white or pink nodules, sometimes pigmented	Typically pigmented (brown/black), may be amelanotic	Translucent or clear; iris pigment epithelial cysts are smooth brown in appearance	Diffuse or multifocal pale infiltration
Vascularisation	Neovascularisation is common	Variable, may show intrinsic vessels	Absent	May show prominent vascularity
Slit lamp findings	Nodules with irregular surface, possibly hyphaema	Nodular or diffuse mass, often pigmented	Smooth cystic structure	Diffuse iris thickening
Ultrasound biomicroscopy (UBM)	Solid irregular mass with moderate homogenous reflectivity, internal hyporeflective spaces	Homogeneous solid mass	Cystic anechoic structure	Diffuse hypoechoic infiltration
Anterior segment OCT	Irregular thickening and nodules	Non‐homogenous thickening, can have posterior shadowing	Well‐defined cystic space	Diffuse infiltration
Systemic association	Usually associated with known primary malignancy, in one third of cases no known malignancy	Mostly primary ocular tumour	Benign	May be associated with systemic lymphoma

### 3.3 Ciliary Body Metastasis

Patients with a ciliary body metastasis present with subtle and nonspecific symptoms such as blurred vision, ocular discomfort, photophobia or sometimes pain, especially if secondary glaucoma develops [9, 48]. Examination findings can include shallowing of the anterior chamber, localised lens displacement, or elevated intraocular pressure secondary to angle closure. Because of the posterior location of the ciliary body, direct visualisation on slit‐lamp examination is often limited, and involvement is therefore frequently deduced from associated signs [15]. Ultrasound B scan or UBM and anterior segment optical coherence tomography (AS‐OCT) can further characterise the extent and location of the lesion. Orbital MRI may show a well‐defined, enhancing intraocular mass at the ciliary body region [49]. Systemic imaging and biopsy may be necessary for definitive diagnosis, especially in patients without a pre‐existing malignancy.

### 3.4 Optic Disc Metastasis

On fundoscopy, optic disc metastases appear as diffuse enlargement of the optic disc in the majority of cases and as a distinct nodule in 16% of cases [50]. Optic disc metastases are unilateral and associated with adjacent choroidal metastases in the majority of cases (74%) [50]. Optic disc oedema, buried optic disc vessels and splinter haemorrhages are other common signs. The differential diagnosis of optic disc metastases includes papilledema, which is usually bilateral, optic neuritis, which is typically associated with visual loss and pain, optic disc drusen, optic disc granulomas and primary tumours of the optic disc head [51]. Ophthalmoscopic features, ultrasonography, OCT of the optic disc, and systemic associations can help distinguish metastatic lesions to the optic disc.

### 3.5 Vitreous Metastasis

Vitreous metastases are rare and are usually associated with cutaneous melanoma [52, 53, 54]. Francis et al. presented 14 eyes of 11 patients with metastatic cutaneous melanoma to the vitreous. Ten out of the 11 patients were treated with immune checkpoint inhibitors (ICIs). Half of the affected eyes showed amelanotic vitreous debris, while five eyes demonstrated an elevated intraocular pressure and four eyes showed a retinal detachment. The vitreous metastatic disease was associated with metastatic disease in the central nervous system in six patients [52].

### 3.6 Retinal Metastasis

Retinal metastases are rare manifestations of ocular metastatic disease. They typically appear as patchy or punctate retinal infiltrates or an elevated retinal mass [55]. The most frequent primary tumour sites are cutaneous melanoma (36%) followed by lung (23%), gastrointestinal tract cancer (17%) and breast cancer (12%) [55].

Multimodal imaging can highlight distinct features of retinal metastasis. On OCT, lesions are hyperreflective and may be associated with subretinal fluid, intraretinal fluid or hyperreflective intraretinal dots, vitreous seeding and choroidal invasion [56]. FFA and ICGA reveal early hypofluorescence and late hyperfluorescence with filling of intrinsic dilated vessels [56, 57]. Ultrasonography shows a medium‐high reflective dome‐shaped tumour [55].

The differential diagnosis for retinal metastasis includes infectious retinitis (viral, bacterial or fungal), primary intraocular lymphoma (which can present as vitreous and retinal infiltrates), choroidal metastasis (typically a deeper, dome‐shaped subretinal mass better visualised and differentiated on OCT), retinal hemangioma, nerve fibre layer infarction, posterior scleritis, and other inflammatory or neoplastic conditions which can be distinguished based on differences in imaging and clinical history. A high degree of suspicion is needed especially in patients with a known systemic malignancy to differentiate retinal metastasis from these mimickers, with biopsy sometimes necessary to confirm the diagnosis [57].

### 3.7 Lens Capsule Metastasis

Lens capsule metastases are rare intraocular metastases that develop secondary to cutaneous melanoma or secondary spread following treatment for iridociliary melanoma [8, 58, 59, 60]. They can appear as pigment deposits in the anterior or posterior lens capsule originating from cutaneous melanoma [58, 59, 60]. Lens capsule melanomas can be accompanied by metastatic spread in the angle and enucleation is a reasonable treatment option [58, 59, 60].

## 4 Management Strategies

The management of intraocular metastasis is nuanced and primarily dictated by factors such as the patient's overall systemic condition, the number and distribution of metastatic lesions, their anatomical location within the eye and whether the involvement is unilateral or bilateral. Systemic workup is usually needed even if there is a known history of cancer, particularly looking for intracranial metastases by neuroimaging. Observation may be recommended for patients whose systemic status is poor or when life expectancy is limited as intervention may not yield significant benefit. If there is non‐ocular metastatic involvement in addition to the ocular deposits, then it is reasonable to start systemic treatment as advised by a medical oncologist and judge the response in the eyes after a few cycles are administered.

For multifocal and bilateral metastatic lesions, systemic therapies such as chemotherapy or immunotherapy are considered appropriate to address widespread ocular involvement and optimise quality of life [61]. When a choroidal metastasis is solitary, especially in patients with good systemic health, targeted local therapies are possible. These include external beam radiotherapy (EBRT), plaque radiotherapy, proton beam radiotherapy (PBRT), gamma‐knife radiotherapy, transpupillary thermotherapy (TTT), or photodynamic therapy (PDT), each offering potential for tumour control while preserving visual function. If there are intracranial metastases, then ocular radiotherapy can be given at the same time.

Enucleation is reserved for cases where the affected eye is blind and painful, providing symptomatic relief when other treatments are ineffective or inappropriate. A multidisciplinary approach involving the medical oncologist is essential to tailor treatment strategies to individual patient needs, balancing ocular prognosis with systemic disease management [62].

### 4.1 Systemic Treatment

The treatment of intraocular metastases with systemic therapies primarily involves managing the underlying primary malignancy using chemotherapy, immunotherapy, hormone therapy, and targeted therapy. The richly vascularised and permeable choroid allows the high penetration of anti‐cancer molecules from the blood [62, 63]. Chemotherapy has been proven to be effective in the treatment of choroidal metastases [5, 64, 65].

Evidence regarding the efficacy of ICIs in intraocular metastases remains limited to case reports and small case series. While some reports describe regression of ocular metastatic lesions following systemic immunotherapy, responses appear inconsistent. Li et al. showed that pembrolizumab (Keytruda)—a PD‐1 inhibitor combined with chemotherapy—induced regression of choroidal metastasis from non‐small cell lung cancer (NSCLC) [66]. Similarly, ipilimumab and nivolumab induced regression of choroidal metastases secondary to renal cell carcinoma in a small case series [67]. However, a recent study using a mouse model of intraocular metastasis has demonstrated that the eye's immune‐privileged microenvironment significantly limits the effectiveness of immunotherapy; specifically, expression of protein biomarkers and inhibitory molecules by ocular tissues was found to impair the function of tumour‐infiltrating T cells [68]. This highlights that while immunotherapy may be effective in select cases, responses are variable and ocular‐specific outcomes remain largely unpredictable. Immune‐related ocular adverse events, such as uveitis and secondary vision loss, may also occur [67].

Hormone‐based therapies such as tamoxifen and aromatase inhibitors target the hormone receptor pathways that drive tumour growth, providing an effective treatment modality for hormone receptor‐positive metastatic breast cancer. Manquez et al. showed regression of uveal metastases from breast carcinoma in 10 out of 17 patients treated with aromatase inhibitors [69]. Furthermore, Matsuo et al. reported a case of a patient with choroidal metastatic disease from prostate cancer treated successfully with degarelix, a gonadotrophin‐releasing hormone (GnRH) antagonist [70]. Recent studies report good choroidal metastatic control in patients with epidermal growth factor receptor (EGFR)—positive and anaplastic lymphoma kinase (ALK) rearranged NSCLC treated with tyrosine kinase inhibitors (TKI) and ALK inhibitors [71, 72].

Antibody–drug conjugates (ADCs) are a new class of cancer treatment. These agents comprise a monoclonal antibody linked to a cytotoxic payload, permitting targeted delivery to tumour cells expressing a specific surface antigen. Upon binding, the cytotoxin is released inducing cell death with limited systemic exposure. ADCs have demonstrated efficacy in metastatic disease, with high objective response rates and durable disease control in tumour types that frequently metastasise to the uveal tract, particularly breast and lung cancers. This makes them highly relevant to the systemic disease settings from which intraocular metastases arise. For example, the HER2‐directed ADC trastuzumab deruxtecan showed marked activity in HER2‐positive metastatic breast cancer, achieving an objective response rate of 79.7% in the DESTINY‐Breast03 trial [73], and in HER2‐mutant NSCLC, with a centrally confirmed objective response rate of 55% and a median progression‐free survival of 8.2 months in DESTINY‐Lung01 [74]. Similarly, sacituzumab govitecan, a Trop‐2‐directed ADC, significantly improved outcomes in metastatic triple‐negative breast cancer, demonstrating an objective response rate of 35% compared with 5% for standard chemotherapy in the phase III ASCENT trial [75].

**FIGURE 2:**
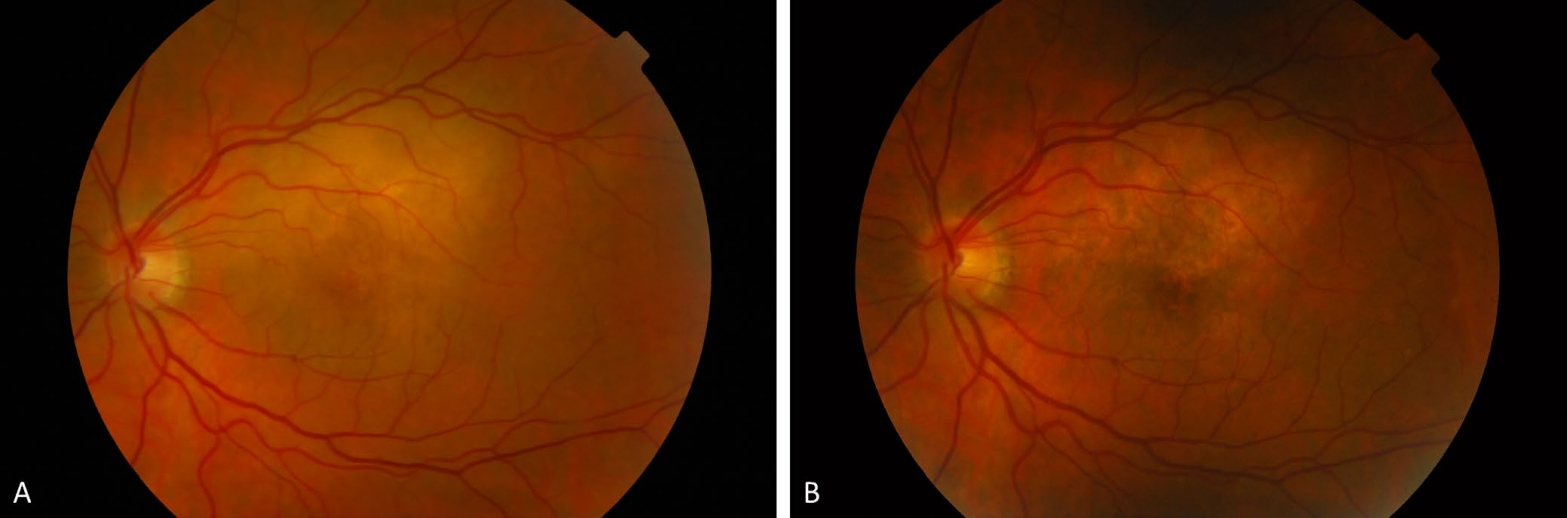
Response of choroidal metastasis to systemic treatment. Colour fundus photograph of HER2‐positive breast cancer metastasis to the choroid of the left eye before treatment (A), treated with systemic docetaxel, pertuzumab, and trastuzumab shows complete regression after four cycles (B). Patient consent obtained for the use of images for publication.

Although these data are derived from systemic disease rather than ocular‐specific assessments, they underscore the potency of ADCs in metastatic contexts that are highly relevant to intraocular metastases. Nevertheless, robust ocular‐specific outcome data for ADCs are currently lacking. Most pivotal ADC trials do not report responses of choroidal or uveal metastases, and published case reports describing intraocular responses are not yet available. Moreover, the blood–retinal barrier shares structural and functional similarities with the blood–brain barrier [76], raising concerns regarding drug permeability. However, growing evidence indicates that ADCs demonstrate clinically significant activity in central nervous system metastases [77], implying potential intraocular penetration. Consequently, the efficacy of ADCs for intraocular lesions remains unquantified, and local ocular therapies remain important. In addition, ADC‐related toxicities may limit their use and necessitate careful systemic monitoring, including myelosuppression and diarrhoea with sacituzumab govitecan, and the risk of interstitial lung disease or pneumonitis with trastuzumab deruxtecan. Ocular toxicity is also a concern, which may limit the use of ADCs for systemic indications. To this end, efforts by Pasricha et al. have led to consensus grading scales for ocular toxicities of emerging oncology treatments, comprising visual acuity, eye symptoms, cornea, conjunctiva/sclera, anterior chamber, and retina/posterior segment adverse events. They have devised a schema for experimental drug dose modification recommendations based on severity [78]. These considerations highlight the importance of multidisciplinary coordination when managing patients with intraocular metastases. Further studies are needed to establish their long‐term benefits, including efficacy to intraocular metastatic disease, and broader applicability [79] (Figure 2).

### 4.2 Photodynamic Therapy (PDT)

**FIGURE 3:**
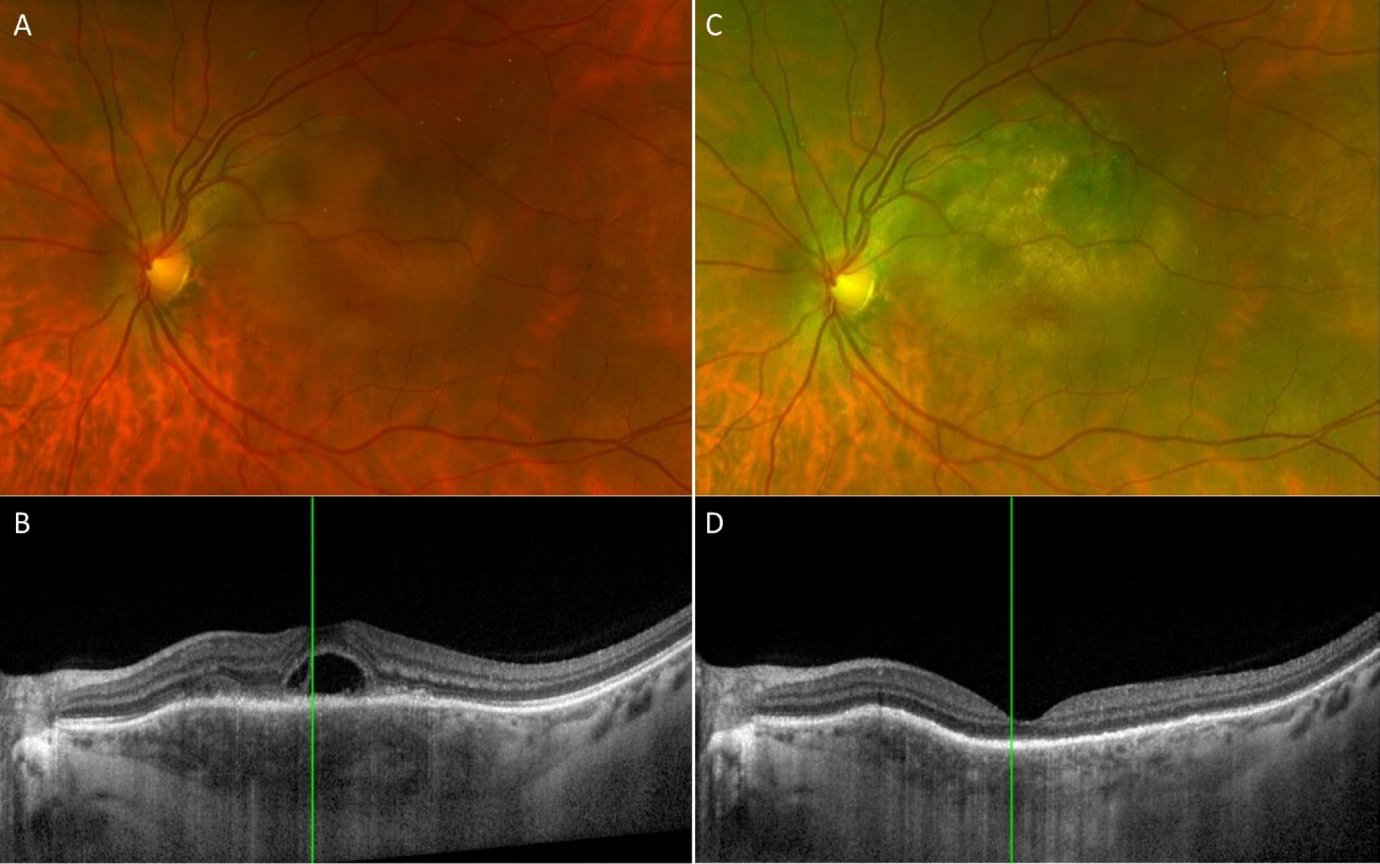
Response of choroidal metastasis to photodynamic therapy (PDT). Pseudocolor image before treatment (A) shows a yellow choroidal lesion and OCT scan (B) shows a bacillary layer detachment (BALAD) and choroidal thickening. Six weeks after double‐duration PDT, pseudocolour image (C) and OCT (D) show good tumour control with resolution of BALAD and improvement of the choroidal thickening. Patient consent obtained for the use of images for publication.

PDT utilises the photosensitiser verteporfin, which is activated by a 689‐nm wavelength laser beam. Upon activation, verteporfin generates reactive oxygen species that induce selective tissue damage and necrosis, primarily targeting abnormal blood vessels in the retina or choroid [80]. This mechanism allows PDT to precisely destroy pathological tissue while sparing the surrounding healthy retina, making it an effective treatment for choroidal metastases by reducing tumour vascularisation and promoting tumour regression. PDT is an effective and well‐tolerated treatment option for choroidal metastasis, particularly for small to medium‐sized tumours [17]. Shields et al. reported tumour control in 78% of 58 patients with small to medium sized choroidal metastases treated with PDT [81]. Ghodasra et al. showed an improved or stable visual acuity in 69% of eyes with choroidal metastasis treated with PDT and a mean decrease in the thickness of the metastases by 0.83 mm [82]. Based on the above, PDT represents a valuable component in the multidisciplinary management of choroidal metastases, particularly in unilateral metastases of small or medium thickness. It is also useful when there is metastatic regrowth after other treatments such as external beam radiotherapy (Figure 3).

### 4.3 Radiotherapy

#### 4.3.1 External Beam Radiotherapy (EBRT)

**FIGURE 4:**
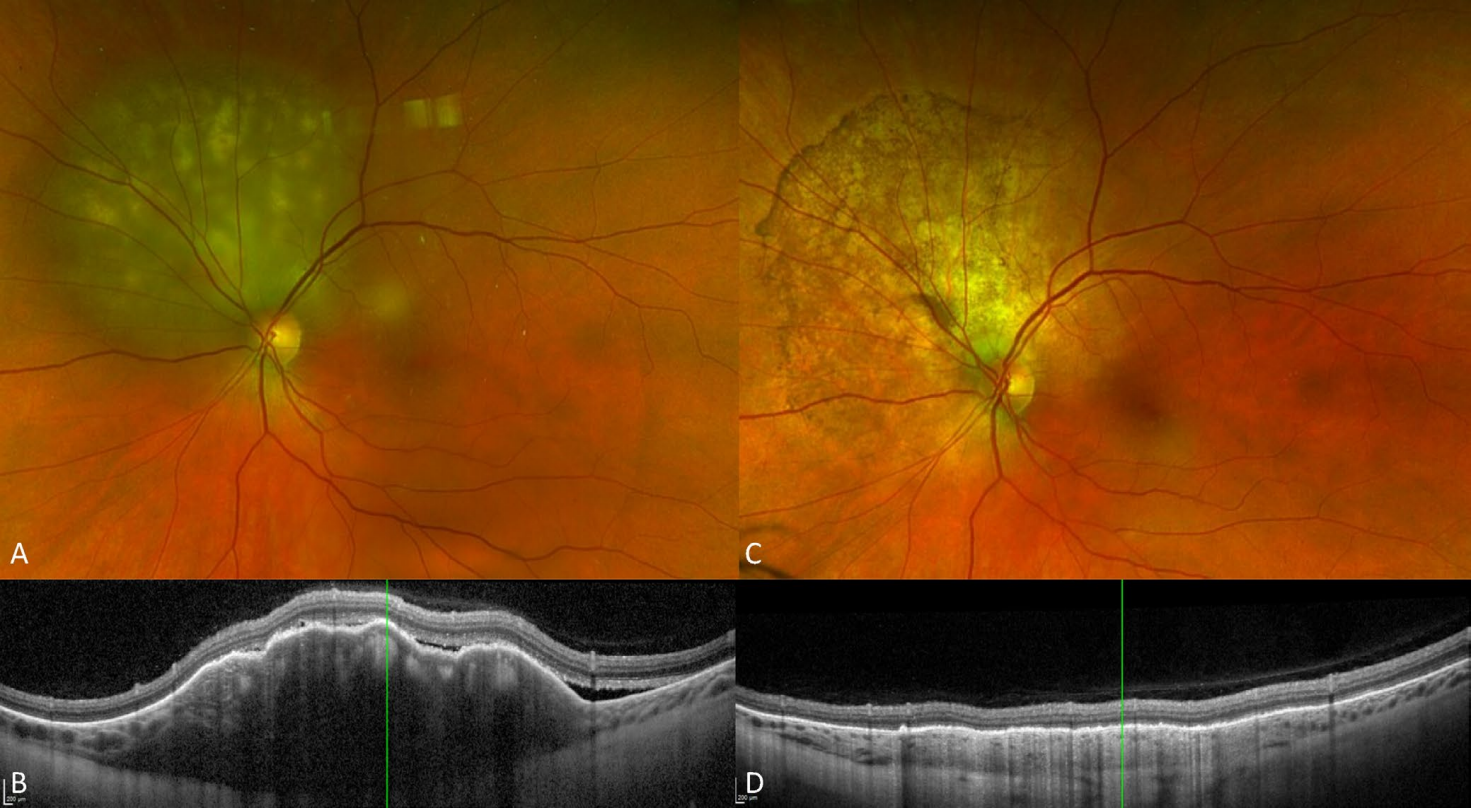
Response of choroidal metastasis to external beam radiotherapy. Pseudocolor image before treatment (A) shows a pale white choroidal lesion and OCT scan (B) shows choroidal thickening with lumpy bumpy appearance and overlying subretinal fluid. Eight weeks after external beam radiotherapy, pseudocolour image (C) and OCT scans (D) show good tumour control with resolution of subretinal fluid and improvement of the choroidal thickening. Patient consent obtained for the use of images for publication.

EBRT is a well‐established and effective palliative treatment for choroidal metastases, typically delivered in fractions. Planning of EBRT involves identifying the metastasis, or gross tumour volume (GTV), on CT imaging. Fundus photography and high‐resolution MRI can aid in more accurate delineation of tumour boundaries. The clinical target volume (CTV) encompasses areas at risk of microscopic invasion surrounding the GTV, typically by applying a 5 mm margin. To account for positional and technical uncertainties, an additional circumferential margin of 3–5 mm is added to define the planning target volume (PTV). Treatment is delivered using a lens‐sparing technique. Commonly used regimens include 30 Gy in 10 fractions or 20 Gy in five fractions [83]. Studies have shown EBRT achieves tumour regression in the majority of cases with visual acuity improvement or stabilisation [83, 84]. While EBRT's extended treatment duration can be inconvenient, especially for patients with poor systemic health, its benefit in maintaining vision and controlling ocular tumour burden often outweighs these disadvantages. Reported adverse effects include cataract formation in approximately 7% of patients, radiation retinopathy in around 3%, exposure keratopathy in 3%, optic neuropathy in 2%, and neovascularisation of the iris in about 2% [84] (Figure 4).

#### 4.3.2 Plaque Brachytherapy

**FIGURE 5:**
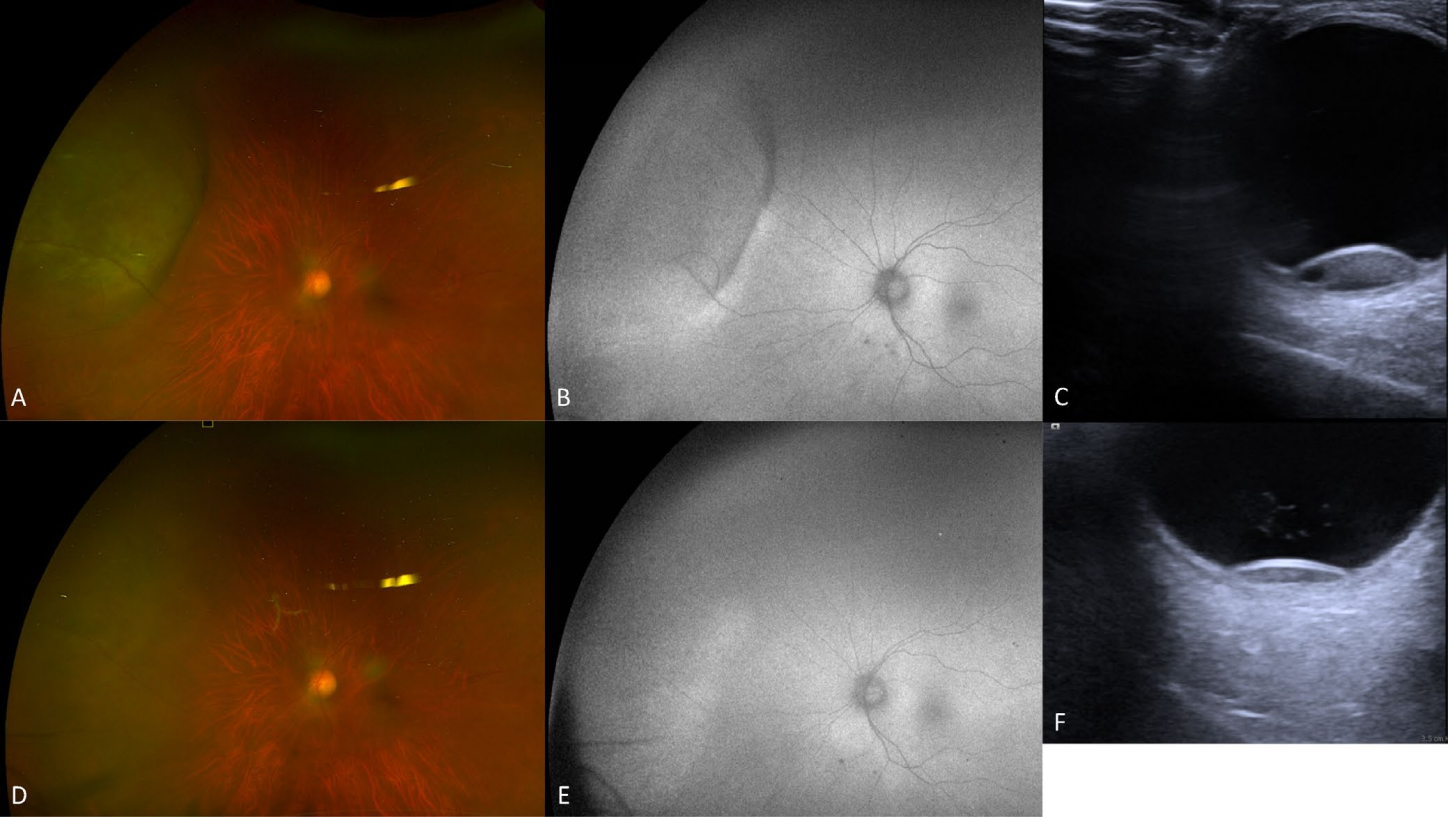
Response of choroidal metastasis to plaque brachytherapy. Pseudocolor image before treatment (A) shows an orange choroidal lesion surrounded by subretinal fluid, as also seen on the autofluorescence image (B). On high resolution ultrasound (C), it has low to medium internal echogenicity, with an echolucent cavity present within the lesion at the nasal border, and 3.4 mm elevation. Three months after the treatment, pseudocolour (D) and autofluorescence images (E) show good tumour control with improvement of the elevation to 1.5 mm (F). Patient consent obtained for the use of images for publication.

Plaque radiotherapy is an effective localised treatment for selected cases of choroidal metastasis, especially solitary lesions. This modality involves the surgical placement of a radioactive plaque on the sclera overlying the tumour, delivering a high radiation dose directly to the metastatic lesion while sparing surrounding healthy ocular structures. Shields et al. studied 36 patients with uveal metastases. In 75% this was the first treatment and in 25% this was a secondary therapy after failure of the uveal tumour to respond to EBRT, chemotherapy, or hormonal therapy [85]. The treatment was effective in 94% of patients during the follow up. Lim et al. confirmed the shrinkage of the metastatic tumour with no acute toxicity [86]. Plaque brachytherapy is relatively short compared to EBRT, often lasting just a few days, which is advantageous for patients with limited life expectancy. Radiation‐related complications such as radiation retinopathy, optic neuropathy, and cataract have been reported but occur at a relatively low rate when compared to the benefits [85, 86]. Plaque radiotherapy can also be effective as salvage therapy in eyes where other treatments, like EBRT or systemic therapies, have failed [85]. Thus, plaque radiotherapy offers a focused, vision‐sparing option within the multidisciplinary management of choroidal metastases, particularly for patients with localised disease and adequate systemic control (Figure 5).

#### 4.3.3 Proton Beam Radiotherapy (PBRT)

PBRT is a precise localised treatment for choroidal metastases, offering high local tumour control while preserving the eye. This modality uses charged proton particles that deliver a concentrated radiation dose directly to the tumour with minimal exposure to surrounding healthy ocular structures. Clinical outcomes show that PBRT achieves tumour regression in the majority of treated cases, with many patients experiencing stabilisation or improvement in visual acuity [87]. Treatment courses are relatively short, which is advantageous for patients with limited life expectancy. Complications include madarosis and lid burns, cataract, iris neovascularisation, and neovascular glaucoma.

#### 4.3.4 Gamma Knife Radiotherapy (GKR)

GKR is a stereotactic radiotherapy option for the treatment of choroidal metastases. It uses focused gamma radiation to deliver a single, high‐dose treatment to the tumour with steep dose gradients that minimise exposure and damage to surrounding healthy ocular structures [88]. Although not yet as widely employed as EBRT or plaque brachytherapy, GKR offers several advantages, including a shorter overall treatment time and reduced radiation‐related complications. Cho et al. recently reported that GKR was safe and effective in seven patients with choroidal metastases [89]. Radiation‐related complications with GKR are generally infrequent but may include cataract progression, transient ocular pain, or increased intraocular pressure [88, 89, 90]. Overall, emerging data support the role of GKR as an alternative or adjunct to established therapies within personalised ocular oncology care.

### 4.4 Transpupillary Thermotherapy (TTT)

TTT is a minimally invasive, localised treatment modality used for small choroidal metastases. It involves the application of an 810 nm diode infrared laser through the pupil, using a contact lens, to deliver controlled heat to the tumour tissue. A previous study showed a tumour regression in 71% of the 59 eyes treated with this modality [91]. Kiratli et al. confirmed that TTT is an effective treatment reporting regression of six out of seven small choroidal metastases with thickness less than 3.5 mm [92]. However, TTT should be applied with caution for tumours near the fovea or optic nerve head, where thermal damage can cause permanent visual acuity loss.

### 4.5 Anti‐Vascular Endothelial Growth Factor (AntiVEGF) Agents

The use of intravitrealanti‐VEGF agents has emerged as a complementary treatment option in managing intraocular metastases, particularly for cases complicated by exudative retinal detachment or macular oedema. Yasui et al. reported a case of exudative retinal detachment secondary to metastatic choroidal tumour from lung adenocarcinoma treated effectively with systemic chemotherapy and intravitreal bevacizumab [93]. Anti‐VEGF therapies can reduce tumour‐associated vascular permeability and neovascularisation, thereby improving retinal anatomy and stabilising or enhancing visual function [94]. While not curative, anti‐VEGF injections have demonstrated efficacy in symptom control and may serve as an adjunct to systemic chemotherapy or localised radiotherapy [95]. Published case series and reviews indicate a potential to delay vision loss, especially when integrated within a multidisciplinary treatment plan [94, 96].

### 4.6 Surgical Intervention

Local resection is rarely indicated for ocular metastases but may be considered in select cases. Surgical excision can be performed in patients with iris or choroidal metastases when the lesions are accessible and sufficiently localised—often using iridectomy/iridocyclectomy for iris/ciliary body involvement [42, 97] and local resection for choroidal lesions [98]. Vitrectomy may help improve vision and comfort in patients with vitreous metastases, and in cases of retinal metastasis [99], both vitrectomy and retinectomy have been reported as surgical options [100, 101, 102]. Enucleation may be required for extensive disease or an irreversibly blind, painful eye, most commonly in association with persistent tumour growth and neovascular glaucoma, when globe preservation is not feasible [42, 57, 60].

## 5 Survival After Ocular Metastasis

Survival in patients with intraocular metastases is closely linked to the primary malignancy, systemic tumour burden, and response to systemic therapy rather than the ocular metastases alone. Shields et al. (2018) reported significant survival differences based on primary cancer type. Patients with breast cancer metastases generally exhibit a more favourable prognosis, with a 5‐year survival of 25%, likely reflecting both the natural biology of breast tumours and advances in targeted treatments including hormone therapy and HER2‐directed agents. By contrast, lung cancer patients tend to have poorer outcomes, with a 5‐year survival of 13% [9]. Notably, pancreatic cancer exhibited the worst survival among primary tumours. Additional parameters influencing survival include laterality of ocular involvement and gender. Welch et al. found that bilateral uveal metastases were associated with decreased survival compared to unilateral cases, likely due to higher systemic tumour load. Female sex was also reported to be linked to a favourable survival compared to male sex [12]. Future research should aim to better understand the factors that predict survival and to incorporate emerging treatments such as targeted therapies and immunotherapies into patient care, with the goal of improving survival outcomes for those with intraocular metastases.

## 6 Conclusion

The diagnosis of intraocular metastasis poses a significant clinical challenge, particularly in patients without a known history of systemic malignancy. Multimodal imaging and, in select cases, biopsy of suspicious lesions can aid in establishing a definitive diagnosis. A range of therapeutic modalities with encouraging outcomes are now available, allowing for personalised treatment strategies that accommodate individual patient needs and clinical circumstances.

## Funding

The authors have nothing to report.

## Conflicts of Interest

M.S.S. has an advisory role with Aura Biosciences Inc., Ideaya Biosciences Inc., and ReBio Pharma. The other authors declare no conflicts of interest.

## Data Availability

Data sharing not applicable to this article as no datasets were generated or analysed during the current study.
